# Systemic Hematogenous Maintenance of Memory Inflation by MCMV Infection

**DOI:** 10.1371/journal.ppat.1004233

**Published:** 2014-07-03

**Authors:** Corinne J. Smith, Holly Turula, Christopher M. Snyder

**Affiliations:** Department of Microbiology and Immunology, Jefferson Medical College, Kimmel Cancer Center, Thomas Jefferson University, Philadelphia, Pennsylvania, United States of America; La Jolla Institute for Allergy and Immunology, United States of America

## Abstract

Several low-grade persistent viral infections induce and sustain very large numbers of virus-specific effector T cells. This was first described as a response to cytomegalovirus (CMV), a herpesvirus that establishes a life-long persistent/latent infection, and sustains the largest known effector T cell populations in healthy people. These T cells remain functional and traffic systemically, which has led to the recent exploration of CMV as a persistent vaccine vector. However, the maintenance of this remarkable response is not understood. Current models propose that reservoirs of viral antigen and/or latently infected cells in lymph nodes stimulate T cell proliferation and effector differentiation, followed by migration of progeny to non-lymphoid tissues where they control CMV reactivation. We tested this model using murine CMV (MCMV), a natural mouse pathogen and homologue of human CMV (HCMV). While T cells within draining lymph nodes divided at a higher rate than cells elsewhere, antigen-dependent proliferation of MCMV-specific effector T cells was observed systemically. Strikingly, inhibition of T cell egress from lymph nodes failed to eliminate systemic T cell division, and did not prevent the maintenance of the inflationary populations. In fact, we found that the vast majority of inflationary cells, including most cells undergoing antigen-driven division, had not migrated into the parenchyma of non-lymphoid tissues but were instead exposed to the blood supply. Indeed, the immunodominance and effector phenotype of inflationary cells, both of which are primary hallmarks of memory inflation, were largely confined to blood-localized T cells. Together these results support a new model of MCMV-driven memory inflation in which most immune surveillance occurs in circulation, and in which most inflationary effector T cells are produced in response to viral antigen presented by cells that are accessible to the blood supply.

## Author Summary

Herpesviruses persist for the life of the host and must be continuously controlled by a robust immune surveillance effort. In the case of the cytomegalovirus (CMV), this ongoing immune surveillance promotes the accumulation of CMV-specific T cells in a process known as “memory inflation”. We and others have proposed that the ability to induce memory inflation may be an important benefit of CMV-based vaccine vectors that persist within the host and continuously boost the immune response. However, it has been difficult to determine where T cells are encountering CMV in the body, leading to many unanswered questions about the maintenance of this remarkable response. Previous models proposed that T cells encountered viral antigen within lymph nodes and then migrated to other tissues to prevent CMV reactivation. However, we found that the majority of T cells stimulated by CMV were present in circulation, where they could be sustained without the input from T cells localized to lymph nodes. In fact, two of the defining features of memory inflation - inflated numbers and an effector phenotype - were restricted to cells that were exposed to the blood. Thus, we propose that memory inflation during CMV infection is largely the result of immune surveillance that occurs in circulation.

## Introduction

Cytomegaloviruses (CMVs) are ubiquitous, β-herpesviruses that establish lifelong infections in their hosts. CMV causes an acute systemic viral infection, followed by latency in many cells throughout the body. Cells of the myeloid lineage and endothelial cells from many organs have been shown to harbor CMV[1]–[17]. However, the sites of viral latency have not been fully defined, largely because it is extremely difficult to detect the virus during latency. Latent CMV is thought to reactivate in a stochastic manner throughout the body[18], [19]. Thus, keeping CMV asymptomatic requires a robust immune surveillance effort by NK cells and virus-specific CD4 and CD8 T cells[20]. For this reason, immune compromised individuals are at great risk of CMV reactivation[21]. Importantly, CMV-specific CD8 T cells directly suppress viral gene expression during this latent/persistent phase of infection[22] and can, in isolation, control CMV replication[23]–[26].

Because of this ongoing immune surveillance effort, the hallmark of the immune response against CMV is CD8 T cell “memory inflation”, a phenomenon in which T cells specific for certain CMV epitopes stabilize at very high levels in the blood of hosts[27]–[31]. Approximately 5% of all CD8 T cells in the average adult are specific for CMV[32], making these T cell populations the largest to be described in the circulation of healthy adults. Although first described in the context of CMV infections, it is now clear that several low-level persistent viral infections can induce and sustain very large numbers of virus-specific T cells[33]–[39]. In all cases, the majority of these inflationary CD8 T cells have a phenotype that is characteristic of effector cell differentiation [29], [40]–[43] which is consistent with repeated antigen exposure (KLRG-1^pos^, CD127^low^, CD62L^low^)[44], [45]. However, the homeostasis of these unusual responses is still poorly understood. Because CMVs are highly species-specific, human CMV (HCMV) cannot be used in any animal model. Fortunately, the natural mouse pathogen murine CMV (MCMV), establishes a remarkably similar host-pathogen balance and promotes robust memory inflation (reviewed in [46]), making this an excellent model.

The robust CD8 T cell response elicited by CMV has also led to its exploration as a vaccine vector against heterologous infections and cancer[47]–[49]. Surprisingly, we have shown that a spread-defective vaccine strain of MCMV (ΔgL-MCMV) was able to induce memory inflation when administered systemically [50], potentially alleviating safety concerns that would arise with a spread-competent vaccine. Importantly, spread-defective MCMV did not induce memory inflation when administered as a footpad injection, indicating that the route of infection (and therefore the site of latency) is of critical importance for memory inflation. A better understanding of where T cells interact with persisting virus is necessary for the effective use of CMV as a vaccine strain.

As might be expected, sustaining effector-phenotype inflationary T cells depends on viral antigen. In humans, the number and phenotype of HCMV-specific T cells directly correlates with peak viral loads[51]. In mice, we and others have shown that division of MCMV-specific inflationary T cells at steady-state occurs only in the presence of antigen [43], [52]. However, we have also shown that steady-state division of inflationary T cells occurs rarely, even in the presence of antigen, and inflationary T cells die with a half-life of approximately 2 months[43]. This half-life is remarkably similar to the half-life of HCMV-specific T cells found in people [53]. We interpreted these data to suggest that inflationary effectors must be continuously replaced from a subset of more proliferative cells. Notably, a minor subset of inflationary T cells retains a memory phenotype (KLRG-1^neg^, CD127^pos^), and these cells seem to be much more proliferative([52] and unpublished data).

Recent work has shown that memory inflation in mice depends on the presentation of viral antigen by non-hematopoietic cells[52], [54]. Moreover, the Oxenius lab found that MCMV-specific T cells in lymph nodes had an elevated rate of division at steady-state, and that these T cells were much more likely to retain a memory phenotype than cells elsewhere in the body[52]. Together these data have led to the hypothesis that reservoirs of viral antigen and/or latently infected non-hematopoietic cells in lymph nodes are responsible for stimulating lymph node-localized memory T cells. These memory T cells are then postulated to divide, producing new effector progeny that leave the lymph node and transit through the blood as they migrate into non-lymphoid tissues for immune surveillance. We tested this model and found instead, that antigen-dependent division and maintenance of inflationary effector T cells occurred systemically and did not depend on T cell egress from lymph nodes. Rather, we found that the vast majority of inflationary T cells, including those undergoing antigen-driven division, were exposed to the blood supply at steady state. Strikingly, the two defining features of memory inflation - inflated T cell numbers and an effector phenotype - were evident primarily within the blood-exposed inflationary T cells. Together, these data suggest a new model of memory inflation in which effector T cell populations are produced and maintained hematogenously.

## Results

### Inflationary populations are maintained stably over time during MCMV infection, despite the turnover of effector T cells

**Figure 1:**
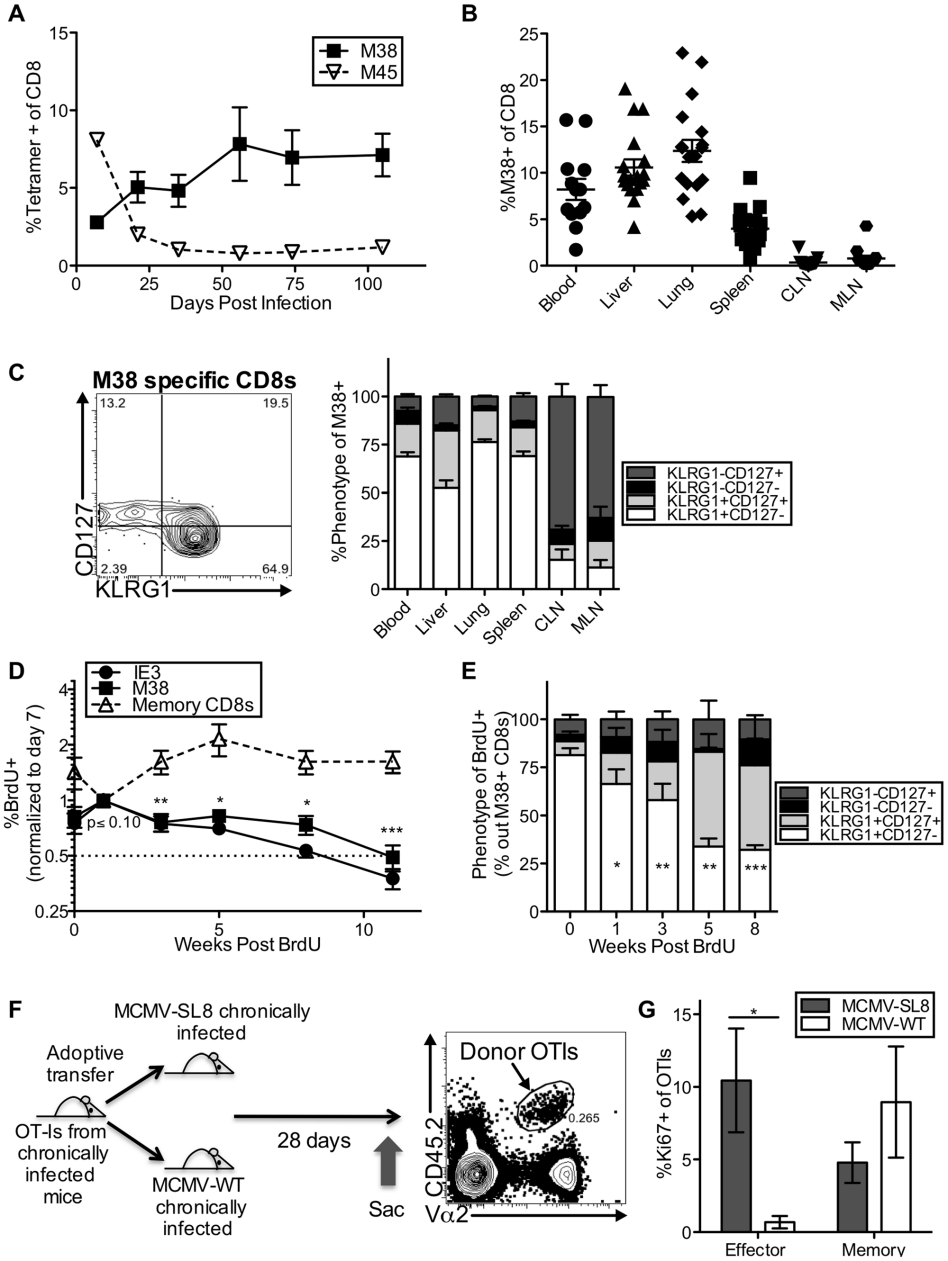
Effector-phenotype CD8s turnover continuously and undergo antigen-dependent division during MCMV-induced memory inflation. (A) In C57BL/6 mice infected with K181 MCMV, M38-specific T cells accumulate over time while M45-specific T cells contract. Shown is the frequency of MCMV-specific CD8s in the blood over time, as measured by tetramer staining. (B) Inflationary T cells are also found in non-lymphoid organs. Shown is the frequency of M38-specific CD8s in the indicated organs of mice infected as in A, more than 3 months post infection. Each symbol represents an individual mouse. (C) Most inflationary T cells express an effector phenotype. Mice were infected as in A. The representative FACS plot (left) shows the KLRG1 and CD127 expression of M38-specific CD8s in the blood. The graph (right) shows the KLRG1 and CD127 expression of M38-specific T cells in organs more than 3 months post infection (n = 12, CLN  =  cervical lymph nodes, MLN  =  mediastinal lymph nodes). (D) Recently divided inflationary T cells are lost over time. B6 mice infected with K181 MCMV or WT-BAC MCMV for more than 3 months were pulsed with BrdU for 3 (n = 6) or 7 (n = 5) days. To account for mouse-to-mouse variation in the incorporation of BrdU, the data was normalized to the frequency of inflationary T cells that were BrdU-positive 7 days post-pulse. Shown is the proportion of M38- and IE3-specific CD8s that retained BrdU in the blood over time. Statistical significance was determined by comparing the proportion of cells retaining BrdU relative to the week 1 time point, using a paired student's t-test (*p<.05, **p<.01, ***p<.001). As a comparison, the same analysis was performed on total CD8 T cells (not antigen-specific) expressing CD127 and lacking KLRG1. Combined data from two independent experiments is displayed. (E) Recently divided inflationary T cells with an effector phenotype are lost over time. Shown is the phenotype of BrdU-positive M38-specific T cells at the indicated time points after BrdU pulse. Any data points with fewer than 25 labeled M38-specific T cells were excluded from the analysis at that time point. Statistical significance was determined as in “D” except that the proportion of BrdU-labeled cells expressing an effector phenotype was compared to the week 0 time point. (F) Adoptive transfer schematic. The representative FACS plot shows donor OT-Is, as a frequency of all CD8s, in the spleen of recipients 28 days after transfer. (G) Donor OT-Is with an effector phenotype only express Ki67 in the presence of cognate antigen. Shown is the Ki67 expression of effector-phenotype (KLRG1^pos^, CD127^low^) and memory-phenotype (KLRG-1^neg^, CD127^pos^) OT-Is in the spleen 28 days post transfer. Results are a combination of two independent experiments (n = 6 per group). Statistical significance was measured by an unpaired student's t-test (*p<.05). In all cases above, error bars represent the standard error of the mean.

The hallmark of the MCMV-specific CD8 T cell response is memory inflation, in which CD8s specific for some epitopes accumulate and remain at high levels for life. In B6 mice, these “inflationary” T cells target peptides derived from the M38, IE3 and m139 proteins (Figure 1A and [28]). In contrast, CD8s specific for epitopes derived from M45 and M57 contract after the acute phase of the infection in a way that resembles a conventional memory response (Figure 1A and [28]). The inflationary CD8s reach high frequencies in the blood, spleen, lungs, and liver of infected mice at late times post infection and the majority have an effector-like phenotype (KLRG1^pos^, CD127^low^, Figure 1B–C for M38, and Figure S2A–B for IE3). In contrast, the frequencies of inflationary T cells remain low in the lymph nodes where the majority retain a memory-like phenotype (KLRG1^neg^, CD127^high^, Figure 1B–C and Figure S2A–B).

Our previous work showed that the inflationary populations in the blood turned over with a half-life of 45–60 days during the latent/persistent stages of infection, even in the presence of viral antigen [43]. Consistent with this, we labeled the inflationary T cells with a brief BrdU pulse and found that the labeled cells - those that divided during the pulse period - decayed over time (Figure 1D), while the total frequency of inflationary CD8s in the blood remained stable during the same time period (Figure S2C). Importantly, the loss of BrdU labeled inflationary cells from the blood paralleled the loss of labeled cells in the spleen, liver and lungs, suggesting that the observed loss of labeled inflationary cells from the blood is not due to migration and accumulation in latently infected tissues (Figure S2D). BrdU-labeled effector phenotype cells were lost much more quickly than non-effector phenotype CD8s of the same specificity (Figure 1E and Figure S2F), even though the phenotype of the overall inflationary populations remained stable over this time (Figure S2E–F).

We and others have shown that MCMV-specific inflationary T cells only undergo extensive proliferation in the presence of antigen[43], [52]. However, it has proven difficult to find viral transcripts at late times post infection, even using an extremely sensitive nested PCR assay (Figure S2G and not shown)[55]. To demonstrate the role that viral antigen plays in the production of inflationary effectors, mice were seeded with OT-Is and infected with MCMV expressing the SIINFEKL peptide from ovalbumin (MCMV-SL8), which induces inflation of SL8-specific T cells including OT-Is[56]. After more than 3 months of infection, CD8 T cells were isolated from the spleens of these mice and adoptively transferred into mice that had been previously infected with MCMV either expressing or lacking SIINFEKL (Figure 1F). Expression of Ki67 by OT-I effector-phenotype cells (KLRG-1^pos^, CD127^low^) was only evident in the presence of antigen (Figure 1G), whereas memory phenotype (KLRG-1^neg^, CD127^high^) OT-Is underwent homeostatic division in both sets of recipient mice. These data show that inflationary effector T cells underwent constant turnover during MCMV infection and that division of inflationary effectors could be used as a read-out of T cell encounter with antigen, even at late times post infection when viral transcripts were undetectable.

### Division of inflationary T cells occurs at an elevated frequency within lymph nodes, but can also be detected systemically

**Figure 2:**
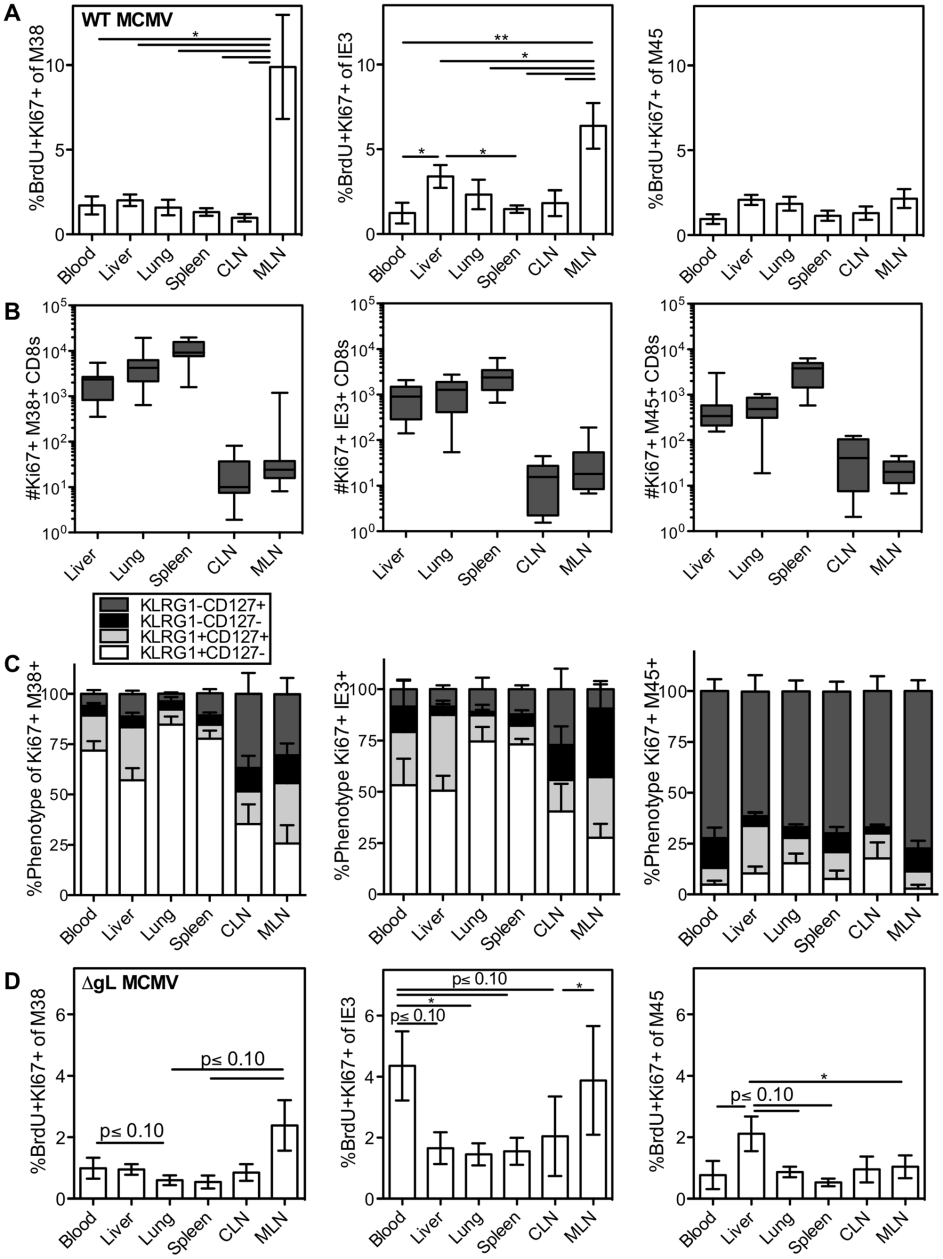
Systemic division of inflationary CD8s. C57BL/6 mice were infected with K181 MCMV for more than 3 months before organs were harvested for lymphocyte analyses. Mice were injected i.p. with 1 mg BrdU 16 hours before sacrifice. (A) Shown is the average frequency of Ki67 and BrdU co-stained M38-specific (left), IE3-specific (middle) and M45 (right) cells in the indicated organs 16 hours after BrdU injection. Error bars represent the standard error of the mean (n = 7). Statistical significance was measured by a paired student's t-test (*p<.05, **p<.01). (B) Number and (C) phenotype of Ki67-expressing T cells specific for M38 (left), IE3 (middle) and M45 (right), in the indicated organs. Error bars represent the standard error of the mean. Data are combined from 3 independent experiments and 12 animals. (D) Spread defective MCMV induces a similar pattern of division as wild type MCMV. C57BL/6 mice were infected with ΔgL-MCMV for more than 3 months and then injected with BrdU as in “A”. Data are displayed as in “A” and combined from two independent experiments (n = 8) Statistical significance was measured by a paired student's t-test (* p<.05).

Current models propose that, to sustain such large effector T cell populations, reservoirs of viral antigen and/or latently infected cells in lymph nodes stimulate T cell proliferation, followed by migration of effector T cell progeny through the blood to non-lymphoid tissues. We assessed cell division during chronic infection by measuring Ki67 expression with or without BrdU incorporation over a short 16 hour time period (gating strategy Figure S1). In agreement with previous work, we found that the division of inflationary cells was elevated in the lymph nodes, although there was a high degree of mouse-to-mouse variability (Figure 2A and Figure S3A). However, this was only evident in the mediastinal lymph nodes (MLN), which drain an i.p. infection ([57] and Figure S2G) and not the cervical lymph nodes (CLN). In contrast, non-inflationary CD8s showed no increase in division in the MLN, suggesting an antigen-specific phenomenon (Figure 2A, third panel). It is important to note however, that the absolute number of inflationary T cells dividing outside of the lymph nodes was much higher than the number dividing within the lymph nodes (Figure 2B). Interestingly, there was a slight, but significant increase in the frequency of dividing inflationary T cells in the liver (Figure 2A and Figure S3A), which is noteworthy because liver sinusoidal endothelial cells are one of the few identified cellular sites of viral latency[58].

In all cases, the dividing inflationary cells in the blood, spleen, liver and lung were primarily effector phenotype, indicating that these dividing cells had recently responded to viral antigen (Figure 2C, M38- and IE3-specific T cells). In the lymph nodes, dividing inflationary cells were less likely to express an effector phenotype than cells in other organs (Figure 2C), but were still skewed away from a memory phenotype (compare Figure 2C to Fig 1C). Notably, a similar anatomical distribution and pattern of division was seen within OT-Is driven to inflate by MCMV-SL8 infection (not shown), indicating that a single T cell clone can display the breadth of phenotype and anatomical distribution induced by memory inflation. In contrast, non-inflationary cells undergoing division in all sites were mostly memory phenotype (Figure 2C, M45-specific T cells).

After an i.p. injection of MCMV, the mediastinal lymph nodes, spleen and liver constitute the first sites of viral infection[57]. We hypothesized that a single-cycle virus, which would be restricted to these first sites, would induce a far more restricted pattern of antigen-dependent effector T cell division. To test this, we used a spread defective ΔgL-MCMV, which induces memory inflation after i.p. inoculation[50]. Interestingly, the pattern of antigen-driven division (Figure 2D and Figure S3B–-C) mirrored that seen after wild-type MCMV infection, with the exception that IE3-specific CD8s had an unusually high rate of division in the blood (compare Figure 2D to 2A). The fact that dividing effector cells were evident in all of the organs was unexpected given that the spread defective ΔgL-MCMV is limited to cells encountered in the first round of infection. Together, the results from both wild-type and ΔgL-MCMV infections lead to two possible interpretations: (i) that T cells stimulated in lymph nodes expand markedly (∼100 to 1000 fold) and migrate into non-lymphoid sites within a short period of time (less than 16 hours) or while continuing to go through the cell cycle, or (ii) that many T cells respond to viral antigen and divide outside of the lymph nodes.

### Division of inflationary effector T cells does not depend on antigen recognition within lymph nodes

**Figure 3:**
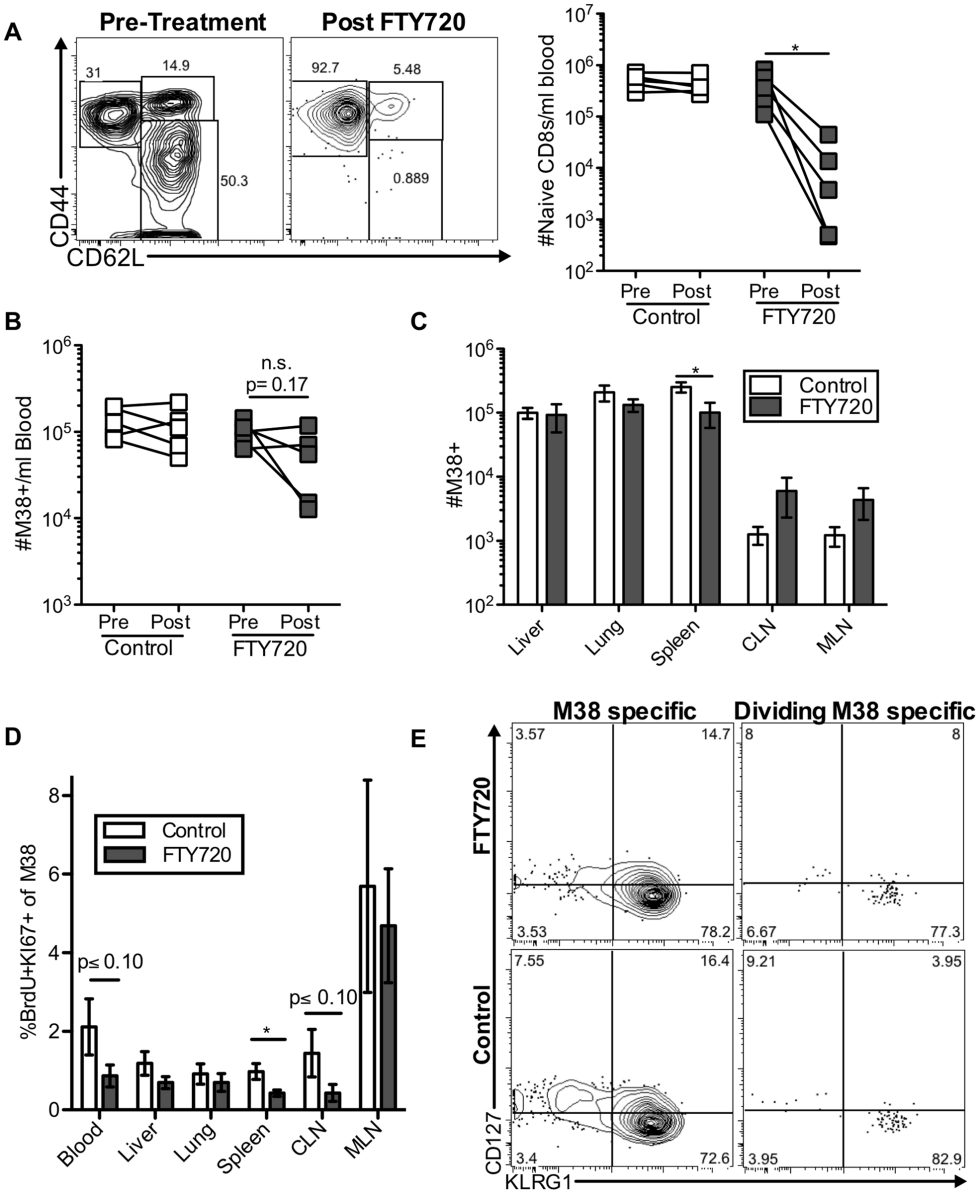
Short-term FTY720 treatment does not alter the pattern of division of inflationary cells. Mice chronically infected with K181 were treated with FTY720 for one week and then injected with BrdU 16(A) Naïve T cells are lost from the blood during FTY720 treatment. The representative FACS plots show the expression of CD44 and CD62L on blood-localized CD8 T cells before (left) and after (right) FTY720 treatment. The graph shows the number of naïve CD8s per milliliter of blood in control and treated mice before and after the treatment. Each line represents an individual mouse (n = 5 per group). (B) M38-specific T cells are only slightly reduced with FTY720 treatment in some mice. Shown is the number of M38-specific CD8s per milliliter of blood in control and treated mice before and after the treatment period. (C) FTY720 treatment has a minimal effect on M38-specific CD8 T cells associated with various organs. Shown are the absolute numbers of M38-specific CD8 T cells harvested with the indicated organs of control (white) and FTY720 treated (grey) mice. (D) Dividing M38-specific T cells are detected systemically even after FTY720 treatment. Shown is the frequency of M38-specific CD8s associated with the indicated organs that were Ki67-positive and labeled with BrdU. (E) Dividing M38-specific T cells are still predominantly effector phenotype. The representative FACS plots show KLRG1 and CD127 expression of M38-specific T cells (left panels) and the dividing M38-specific population in the blood (right panels) for FTY720 treated mice (top panels) and control mice (bottom panels). Data is pooled from two independent experiments (n = 5 per group). In all cases, error bars represent the standard error of the mean. Statistical significance was measured by paired (A and B) or unpaired (C and D) student's t-test (*p<.05).

To directly test whether the division of inflationary T cells depends on antigen recognition within the lymph nodes, mice infected with MCMV for more than three months were treated with FTY720, a drug that blocks lymphocyte egress from the lymph nodes[59] and may force the retention of cells within the parenchyma of tissues[60]. Within one week of treatment, naïve CD8 T cells were significantly reduced in the blood of all mice as expected (Figure 3A), leaving mostly CD44^hi^, CD62L^lo^ cells in circulation. In contrast, the impact of FTY720 on MCMV-specific inflationary CD8 T cells - the vast majority of which are CD44^hi^ CD62L^lo^ (Figure S4A) - was minimal. In the blood, the numbers of inflationary CD8 T cells were reduced in some but not all mice (Figure 3B) and a small, but significant reduction in inflationary CD8 T cell number was evident in the spleen (Figure 3C). However, there were no other significant changes in the numbers of inflationary T cells elsewhere in the animal (Figure 3C). Even more remarkably, the pattern of inflationary T cell division throughout the animal was largely unchanged and dividing cells were still detected at all sites, albeit with slightly reduced frequencies in the blood, CLN and spleen (Figure 3D). Importantly, the phenotype of the total inflationary population and of the dividing inflationary cells in the blood and other organs was not changed by FTY720 treatment (Figure 3E and not shown).

### Maintenance of memory inflation does not depend on T cell trafficking through and/or egress from lymph nodes

**Figure 4:**
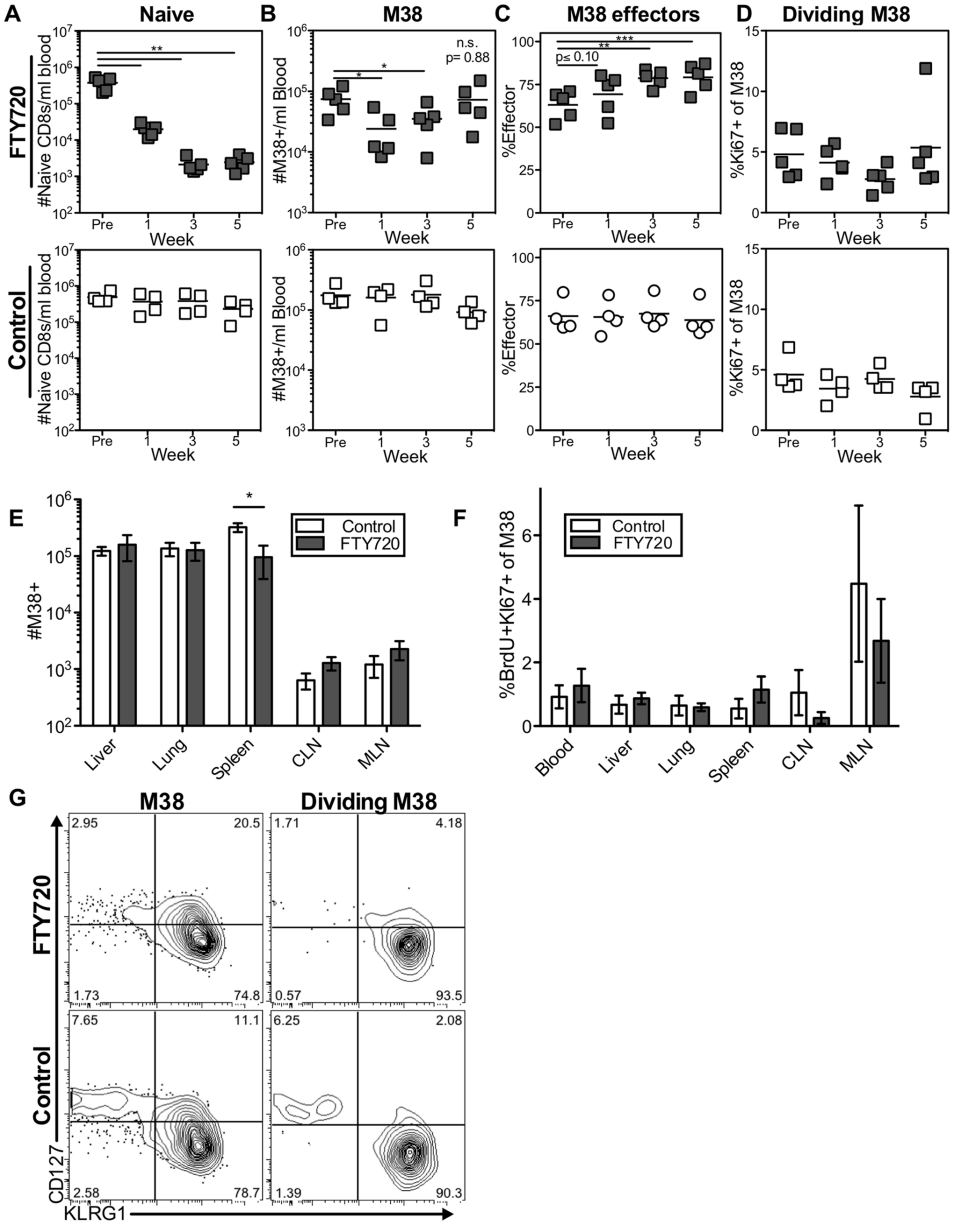
Long-term FTY720 treatment does not alter the maintenance of memory inflation. Mice infected with K181 MCMV for more than 3 months were treated with FTY720 in their drinking water for five weeks. Mice were injected with BrdU 16(A–D) The M38-specific T cell population is stable in the blood of mice during prolonged FTY720 treatment. Shown is (A) the number of naïve CD8s per milliliter of blood, (B) the number of M38-specific CD8s per milliliter of blood, (C) the percentage of blood-localized M38-specific CD8s expressing the effector phenotype (KLRG1^pos^, CD127^low^), and (D) the frequency of Ki67-positive M38-specific CD8s in the blood before and during the time course in treated mice (top) and controls (bottom). (E–G) Prolonged FTY720 treatment does not alter the number, division, or phenotype of dividing M38-specific T cells associated with the organs. Shown are (E) the absolute numbers of M38-specific CD8s and (F) the frequency of M38-specific T cells that were Ki67-positive and labeled with BrdU in control (white) and FTY720 treated (grey) mice. The representative FACS plots (G) show the KLRG1 and CD127 expression of M38-specific T cells (left panels) and dividing Ki67-positive M38-specific population (right panels) in the blood of FTY720 treated (top panels) and control mice (bottom panels) at the end of the treatment period. In all cases, error bars represent the standard error of the mean (n = 5 per group). Statistical significance was measured by paired (A–D) and unpaired (E–F) student's t-tests (*p<.05, **p<.01, ***p<.001).

The previous data suggest that antigen stimulation within the lymph nodes is not responsible for the majority of the dividing inflationary T cells associated with other organs at any given time. However, there was some reduction in the number of antigen specific cells in the blood and spleen, and a slight reduction in the frequency of dividing cells in some sites upon treatment with FTY720 (Figure 3). These results raise the possibility that inflationary effector T cells might transit through non-lymphoid tissues, and return to the blood after draining back to lymph nodes via lymphatics. Such a migration pattern has been described for effector memory T cells [61]. To test whether prolonged FTY720 treatment would compound the effects observed after one week, mice were treated with FTY720 in the drinking water for five weeks. As expected, naïve cells in the blood declined significantly after one week of treatment, continued declining over the next two weeks, and remained low thereafter (Figure 4A). As shown above, the number of inflationary cells per milliliter of blood was reduced after one week of treatment in some, but not all mice (Figure 4B). Strikingly however those numbers rebounded, and by the last time point there was no difference in the number of inflationary T cells in the blood, liver or lungs (Figure 4B and 4E). Since inflationary effector T cells are produced in an antigen-dependent manner and have shorter half-life than the rest of the inflationary population, any effect of FTY720 on memory inflation should manifest first as a preferential loss of the effector subset. However, we found the opposite to be true. The frequency of effectors among the inflationary populations in the blood increased during prolonged FTY720 treatment (Figure 4C), which could suggest that the memory-phenotype T cells in circulation were being slowly sequestered within lymph nodes. Importantly, there was no change in the frequency of dividing inflationary cells in the blood at any time point during FTY720 treatment (Figure 4D). Comparable results were obtained for IE3-specific T cells (Figure S4B–D).

As in the blood, prolonged FTY720 treatment had no effect on the numbers of inflationary T cells associated with the organs or in the proportion of inflationary T cells undergoing division (Figures 4E and 4F). Importantly, the splenic inflationary T cells, which were reduced after one week of treatment, were not progressively lost with prolonged FTY720 treatment (compare Figures 3C to 4E). Finally, the phenotype of the dividing cells was largely unaltered at any time point in the blood or in any organ at the end of the experiment (Figure 4G and not shown). Although these data do not exclude a role for lymph nodes in the circulation of the inflationary T cell pool, they show that the antigen-dependent maintenance of MCMV-specific effector T cells does not depend on migration through or antigen recognition within the lymph nodes.

### Inflationary cells in the lungs and liver during chronic infection are perfusion resistant cells exposed to the blood

**Figure 5:**
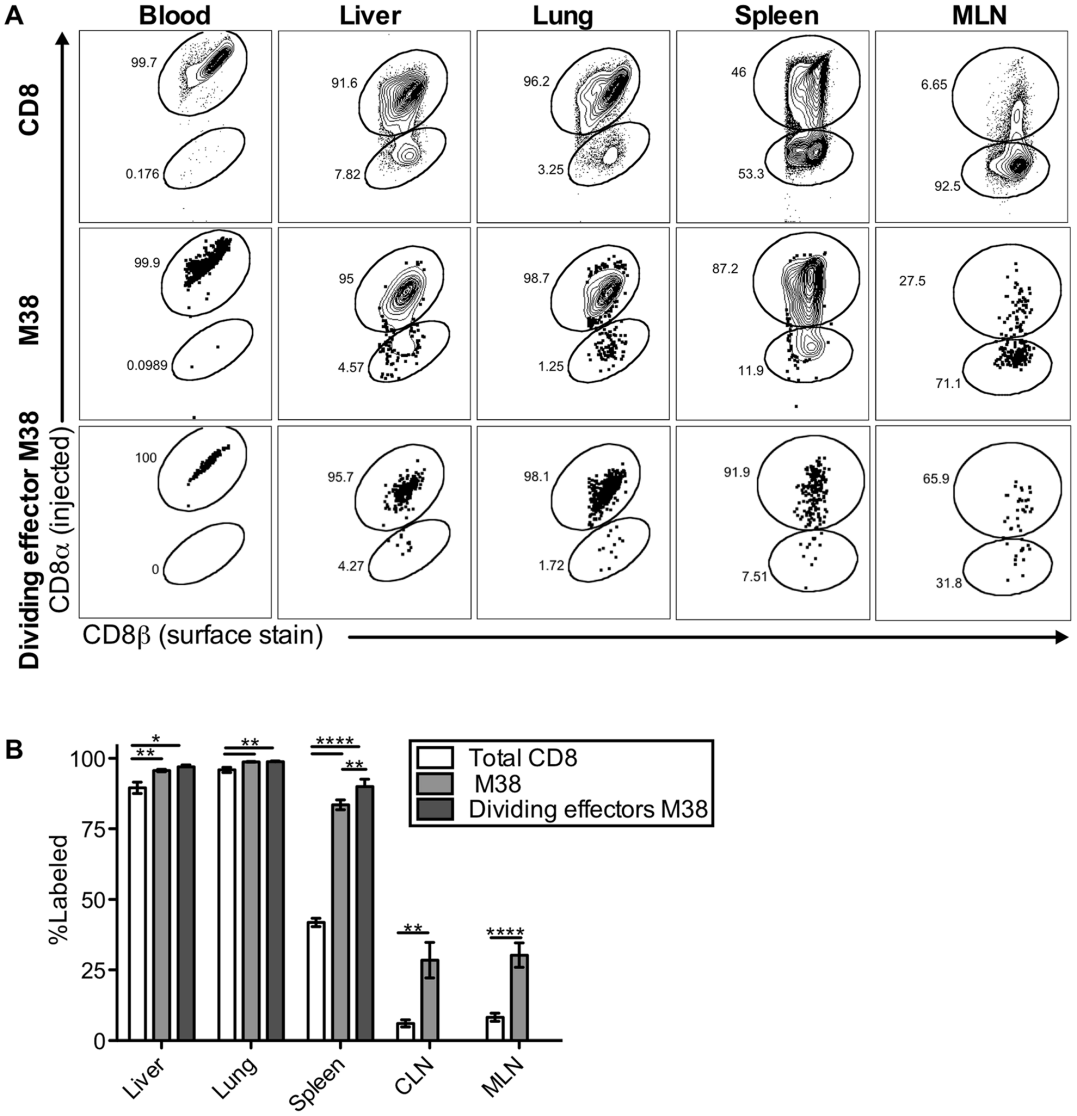
Most inflationary CD8s are exposed to the blood supply. Mice infected with K181 MCMV for more than 3 months were injected with fluorochrome labeled anti-CD8α antibody to identify T cells exposed to the blood supply. After perfusion and processing the organs, cells were counterstained with anti-CD8β, to identify all CD8 T cells. (A) Shown is representative FACS staining with the injected antibody for all CD8s (top), M38-specific CD8s (middle), and dividing effector-phenotype M38-specific CD8s (bottom) in the blood, spleen, liver, lung and mediastinal lymph nodes. (B) Graph shows mean frequency of cells labeled by i.v. staining for all CD8s, M38-specific CD8s, and dividing effector-phenotype M38-specific CD8s for the indicated organs. Data are pooled from 4 independent experiments (n = 15) and representative of seven independent experiments. Statistical significance was measured by paired student's t-tests (*p<.05, **p<.01, ***p<.001, **** p<.0001). Error bars represent the standard error of the mean.

Surprisingly, our data indicate that the maintenance of inflationary effectors does not depend on egress from lymph nodes or the recirculation of inflationary T cells through tissues and back to the blood via lymphatics. This is in contradiction to the current model, which predicts that immune-surveillance against the latent virus depends on the constant migration of inflationary cells from lymphoid to non-lymphoid organs. This led us to ask whether the inflationary cells observed in association with the organs after perfusion (Figures 1–4) had actually migrated into the parenchyma of those organs. To test this, mice infected for more than three months were injected intravenously (i.v.) with fluorescently-labeled anti-CD8α antibody, and sacrificed three minutes later. As in all experiments above, mice were perfused until there was no visible evidence of blood in the target organs. Organs were harvested after perfusion and the harvested cells were co-stained with a CD8β-specific antibody and tetramer. Recent work has shown that this approach can distinguish cells exposed to the blood supply (labeled by the i.v.-injected CD8α-specific antibody) from those that have migrated into the parenchyma of a tissue (labeled only with the CD8β-specific antibody added post-harvest)[62]–[64]. In agreement with previous work, we found that this technique labeled CD8α T cells in the blood, the vasculature of the lungs and lymph nodes, the red pulp of the spleen, and the sinusoids of the liver, which have fenestrated endothelium and are permeable to the blood-borne antibody (Figure 5A top; Figure S5 and [62], [64]). In contrast, CD8s in the white pulp of the spleen, as well as those outside of the lymph node and lung vasculature were unlabeled (Figure S5 and [62], [64]). Strikingly, despite the perfusion, nearly all of the MCMV-specific inflationary cells extracted with the lung and the liver were labeled by the i.v. injected antibody, indicating that these cells were exposed to the blood supply (M38-specific T cells Figure 5A middle, IE3-specific T cells Figure S6D). In the spleen, inflationary T cells were skewed toward the red-pulp, while in lymph nodes, a minority of the inflationary T cells were labeled, as expected. Inflationary T cells in all organs were more likely to be exposed to the blood when compared with the CD8 T cell population as a whole (Figure 5A and 5B) and dividing effector cells were overwhelmingly skewed towards the i.v. labeled fraction in the liver, lung and spleen (Figure 5A bottom row and 5B). Analyses of dividing inflationary T cells in the vasculature of the mediastinal lymph nodes was difficult due to the low cell numbers and high degree of mouse-to-mouse variability. However, in animals with an adequate number of i.v. labeled cells for analysis, a substantial fraction of dividing MCMV-specific T cells were i.v. labeled (Figure 5A bottom row), suggesting that the elevated frequency of T cell division evident in the MLN might be, at least in part, the result of T cells responding to antigen in the vasculature. Together, these data show that inflationary T cells associated with the lung and liver are almost all perfusion-resistant T cells residing in the vasculature, while in the spleen, inflationary T cells preferentially localize to the red-pulp. These results suggest a new model of memory inflation in which the vast majority of MCMV-specific T cells that are responding to the virus are exposed to the blood supply, even within secondary lymphoid organs

### The defining characteristics of memory inflation are restricted to blood-exposed MCMV specific CD8s

**Figure 6:**
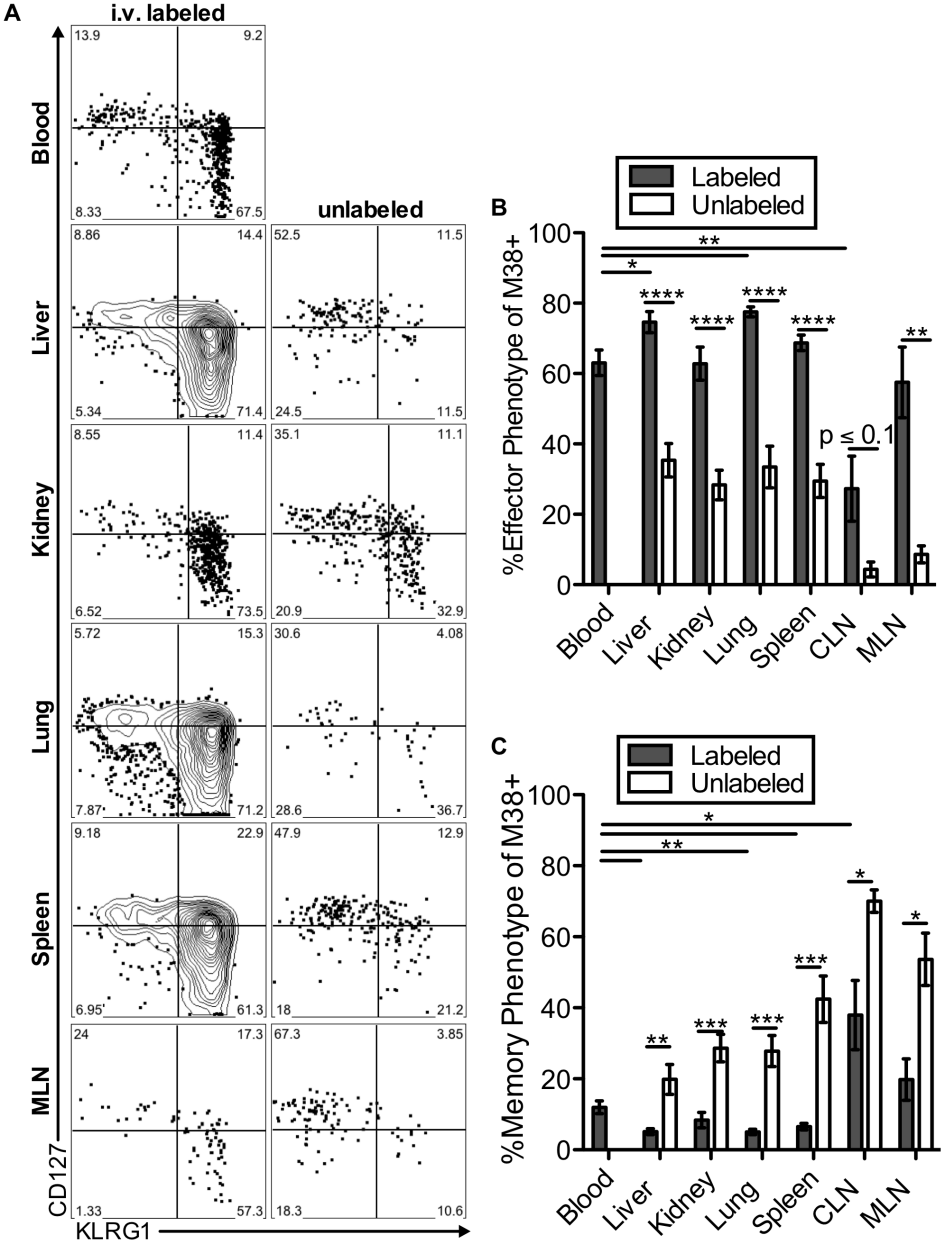
Inflationary cells exposed to the blood are phenotypically distinct from those that are not. Mice infected with K181 MCMV for more than 3 months were injected with fluorochrome labeled anti-CD8α antibody to identify T cells exposed to the blood supply. (A) Representative FACS plots of KLRG1 and CD127 expression on M38-specific CD8s in the labeled (left) and unlabeled (right) fractions of the indicated organs. (B and C) Graphs show the mean frequency of M38-specific cells within the labeled or unlabeled fraction of the indicated organs that express (B) an effector phenotype (KLRG1^pos^, CD127^low^) or (C) a memory phenotype (KLRG-1^neg^, CD127^pos^). Data is pooled from three independent experiments (n = 12). Error bars represent the standard error of the mean. Statistical significance was measured by paired student's t-test (*p<.05**p<.01, ***p<.001, **** p<.0001).

The two primary hallmarks of memory inflation are the numerical dominance of inflating populations and the effector-skewed phenotype of inflationary T cells, both of which are thought to result from repeated antigen stimulation. Thus, we next asked whether these hallmarks were preferentially associated with the blood- or tissue-localized T cell fractions. For this, we included analyses of inflationary T cells associated with the kidney, since the lungs have been the only non-lymphoid organ with a closed circulatory system analyzed to this point. Of note, approximately half of the inflationary T cells associated with the perfused kidney were exposed to the blood supply (Figure S6A). In all organs, cells labeled with the i.v. antibody exhibited the effector-skewed phenotype that is typical of inflationary T cells (M38: Figure 6, IE3: Figure S6B-C). In contrast, unlabeled inflationary cells in all organs were much less likely to express an effector phenotype and were far more likely to exhibit a memory phenotype (Figure 6B–C, Figure S6B–C). Importantly, identical results were obtained with OT-Is undergoing inflation in response to MCMV-SL8 (not shown), indicating that such diversity can be produced by a single T cell clone.

**Figure 7:**
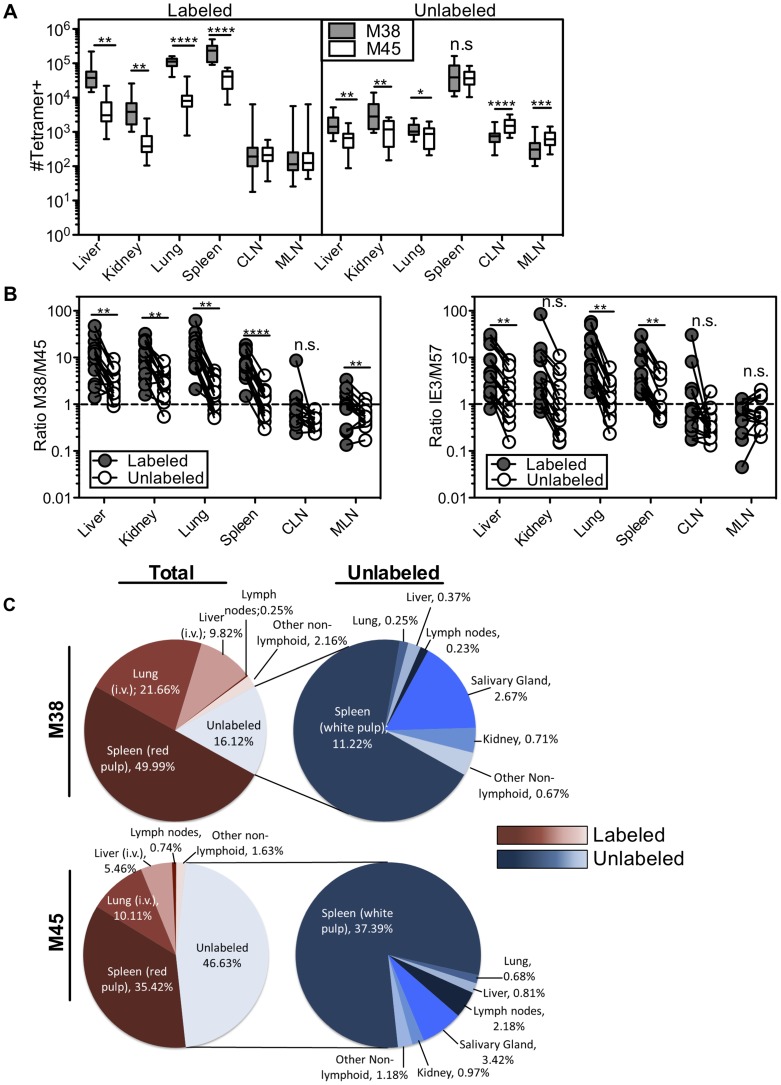
The numerical dominance of inflationary cells is only evident within the blood exposed T cell population. Mice infected with K181 MCMV for more than 3 months were injected with fluorochrome labeled anti-CD8α antibody to identify T cells exposed to the blood supply. (A) Absolute numbers of M38- and M45-specific in the labeled fraction (left panel) and unlabeled fraction (right panel) in the indicated organs. Error bars represent the standard error of the mean. (B) The ratio of M38-specific to M45-specific CD8s was derived from numbers shown in 7A. The left panel compares this ratio in the labeled and unlabeled fractions within each organ. Each line connects the populations found in an individual mouse (n = 15). The right panel shows the same comparison between IE3-specific (inflationary) and M57-specific (non-inflationary) CD8s using numbers derived from Figure S6D. Statistical significance was measured by paired student's t-test (*p<.05**p<.01, ***p<.001, **** p<.0001). (C) A global picture of MCMV-specific CD8 distribution was determined by calculating the average number of tetramer stained cells in the labeled and unlabeled compartments of the spleen, liver, lung, cervical and mediastinal lymph nodes, kidney, female reproductive tract, salivary gland, and mammary gland (n = 7−15). (C) The left panel shows the fraction of the total tetramer positive population that is localized to each site for M38-specific (top) and M45-specific CD8s (bottom). The blood localized fractions for each organ are displayed in red and the unlabeled fractions are displayed in blue. The right panel shows the distribution of only the cells that are shielded from i.v. staining. The numbers represent the percentage of cells at each site out of the total tetramer specific population.

Remarkably, the immunodominance of inflationary T cells also differed between the i.v.-labeled and unlabeled T cells. In the i.v.-labeled fractions of the liver, kidney, lung and spleen, M38-specific T cells were approximately 8 to 16-fold more numerous than non-inflating M45-specific T cells (Figure 7A–B). In contrast, in the unlabeled compartment of the same organs, inflationary M38-specific T cells were only 1.3 to 3-fold more numerous than M45-specific T cells (Figure 7A–B). In fact, M38-specific T cells were subdominant to M45-specific cells in the parenchyma of the lungs in half of the mice, and in the lymph nodes and white pulp of the spleen of most mice (Figure 7B). Importantly, identical results were obtained by comparing the inflating IE3-specific T cell population with the non-inflating M57-specific population (Figure 7B and Figure S6D). Together, these data show that the numerical dominance and the effector phenotype of inflationary T cells, both primary hallmarks of memory inflation and repeated antigen encounter, are almost entirely restricted to T cells exposed to the blood supply.

Using the absolute numbers of recovered T cells, we assessed T cell localization to the vasculature and parenchyma of the spleen, lymph nodes, lungs, liver and kidney as well as other potential sites of T cell migration including the salivary gland, mammary gland and female reproductive tract. When compared to cells within the lung, liver and spleen, T cells associated with the lymph nodes, kidney and mucosal organs were substantially less likely to be labeled by the i.v. staining (Figure S6A). However there were many fewer MCMV-specific cells present at those sites (Figure 7A and Figure S6D). As a result, the vast majority of inflationary cells in an animal were exposed to the blood (Figure 7C). So as not to count circulating cells twice, this analysis does not include cells recovered in the blood itself, which we estimate to be approximately comparable to the number of cells in the spleen (compare Figures 3B and 4B with Figure 7A). Thus, the total fraction of inflationary T cells exposed to the blood is likely to be even greater than that reported here. The preference for blood localization of memory inflation was even more apparent when we analyzed inflationary T cells with an effector phenotype, greater than 90% of which were exposed to the blood (Figure S7A). In marked contrast, approximately half of the non-inflationary cells (Figure 7C) and half of inflationary cells with a memory phenotype were unlabeled by the i.v. antibody (Figure S7B). For both inflationary and non-inflationary T cells, cells that were protected from the i.v. antibody staining were primarily found in the white pulp of the spleen and the salivary gland (Figure 7C). Collectively, these data support a new model of MCMV-driven memory inflation in which the majority of inflationary T cells in the body respond to antigen while remaining exposed to the blood supply. In other words, these data suggest that memory inflation is primarily a blood-localized phenomenon.

## Discussion

Memory inflation is a unique immune response that provides continuous immune surveillance against a lifelong infection without inducing T cell exhaustion[27], [29], [31], [42], [43], [65], [66]. However the mechanism that supports memory inflation has not been well defined. Our previous data support the model that memory inflation during MCMV infection is maintained by systemic antigen-dependent production of short-lived effectors[43]. Here, we show that T cell division originating in the lymph nodes, as well as the migration of T cells through lymph nodes, was completely dispensable for the long-term maintenance of these effector populations. Furthermore, our data show that the bulk of inflationary CD8s that appear to be “in” organs are part of a circulating population with access to the blood, with only a small minority residing within non-lymphoid organs at any given time during the late stages of infection. Indeed, the immunodominance profile and effector phenotype that are characteristic of memory inflation were primarily evident in the blood-localized compartment and not within cells that were shielded from the blood supply. These data lead us to propose a new model of memory inflation in which the effector T cell populations are primarily produced by exposure to antigen that is accessible to the blood supply and are subsequently maintained in circulation.

One of the major setbacks to understanding the maintenance of effector T cells during memory inflation has been the difficulty in defining the sites of ongoing antigen stimulation. It is clear that CD8 T cells suppress viral gene expression during latent infection and that exposure to cognate antigen throughout infection results in T cell division and effector differentiation[22], [43], [52]. We used division of inflationary effectors, which is absolutely antigen-dependent (Figure 1G), to better define the localization and trafficking pattern of T cells produced in response to antigen stimulation. The previous model of memory inflation for both MCMV and adenovirus proposed that antigen depots in the lymph nodes are responsible for stimulating T cells that subsequently migrate into non-lymphoid tissues[33], [52]. Our data do not refute the idea that some antigen recognition occurs in lymph nodes. Indeed, we also observed a clear trend towards more frequent division of inflationary CD8s, but not non-inflationary CD8s, within mediastinal lymph nodes (the draining lymph nodes after i.p. infection, e.g. Figure 2A), clearly suggesting an antigen-dependent process. However, we also observed division of effector T cells associated with all studied organs, where they outnumbered the dividing T cells in the MLN by 100 to 1000-fold (Figure 2C). Most significantly, the proportion of cells dividing at each site was hardly altered by pre-treatment with FTY720, a drug that potently inhibits T cell egress from lymph nodes (Figure 3 and 4). Thus, memory inflation in circulation can be maintained without input from the lymph nodes or cells recirculating through non-lymphoid tissues. Our observation that some, but not all mice exhibited transient decreases in the number and frequency of dividing cells in the blood after FTY720 treatment, might suggest that the lymph nodes contribute T cells to the circulating pool, but that this contribution is dispensable. Alternatively, since very few latently infected cells express viral transcripts at any given time[18], [67], the contribution of any individual site to memory inflation would be expected to vary from mouse-to-mouse and from time-to-time, depending where viral reactivation happened to occur. It is important to note that our FTY720 results do not rule out a role for the spleen in driving memory inflation. In fact, we think it likely that the spleen makes a major contribution to memory inflation due to the large number of inflationary cells found there and the fact that stroma of the spleen is a known site of MCMV latency and reactivation[4]. Because the spleen has an open blood supply, FTY720 would not be expected to prevent the continous circulation of cells through the red pulp as they move from the marginal sinuses to the venous sinuses. On the other hand, it has been suggested that T cell egress from the white pulp (but not the red pulp) is S1PR1-dependent and modulated by FTY720 [68]–[70]. However, trafficking of T cells out of the white pulp hasn't been fully characterized. Determining the precise roles of antigen presentation by specific cells in the spleen and other organs awaits further study.

Our results, which rule out lymph nodes as a necessary source of memory inflation, raise the question of what other sites might be important for driving inflation. Inflationary effector T cells, including those undergoing antigen-driven cell division, were almost entirely exposed to the blood supply, and we have previously shown that their accumulation depends on antigen recognition[56], [71]. Thus, we propose that most cells latently infected with MCMV may also be exposed to the blood supply or accessible to T cells that are in circulation. Although our understanding of the cell types that harbor latent MCMV is incomplete, endothelial cells were previously defined as a major site of MCMV latency[1] and liver sinusoidal endothelial cells (LSECs) were recently identified as a cellular site of both latency and reactivation[58]. Interestingly, LSECs are in direct contact with blood circulating T cells, and can stimulate CD8 T cells[72], and the sinusoids are narrow enough that T cells must crawl through them[73]. Moreover, antigen-presentation by non-hematopoietic cells is critical to sustain inflationary T cell populations[52], [54]. Thus, it is appealing to hypothesize that antigen presentation by sinusoidal endothelial cells is a main driver of memory inflation. Reactivating virus has also been described in the stroma of the spleen[4] (also containing the sinusoids through which circulating T cells must pass), the lungs[19], [67], and the kidney[74] although the specific cell types harboring virus in these organs remain undefined. In addition, CMV-specific T cells express the CX3CR1 and CXCR3 receptors, which attract them to activated endothelial cells expressing fractalkine and IP-10[65], [75]. Thus, it is possible that antigen recognition and the resultant production of effector T cells are almost entirely contained within the vasculature and sinusoids of multiple organs during MCMV infection.

It is tempting to speculate that antigen recognition within circulation is a critical factor in the induction of memory inflation in general. Previous work showed that HSV-1 induced memory inflation when administered systemically, but not after a local infection[34], [76]. Likewise, we showed that a spread-defective vaccine strain of MCMV had to be administered systemically to induce memory inflation[50]. Although we cannot rule out a role for latently infected cells in other compartments, it is possible that memory inflation is so apparent during CMV-infections because it is primarily happening as a result of immune surveillance in the blood. In support of this, two of the defining features of memory inflation - the effector phenotype of the inflationary cells and the immunodominance of CD8s with inflationary specificities - were primarily confined to cells exposed to the blood supply (Figures 5–7). It should be noted that non-inflationary T cells are not thought to recognize viral antigen often, if at all, during the persistent/latent phase of MCMV infection [43], [77], [78]. These non-inflators were co-dominant within the white pulp of the spleen, as well as the parenchyma of the kidney, lung and lymph nodes. The splenic white pulp is known to attract memory-phenotype T cells[79] and CMV-specific T cells within lymph nodes lack any obvious memory inflation [52], [80]. However, non-lymphoid organs were thought to be the targets of the inflationary effector T cells transiting through the blood. Therefore it was surprising that the hallmarks of memory inflation were largely absent from the T cell populations protected from the i.v. antibody staining within non-lymphoid organs.

It is important to note that inflationary CD8s are thought to participate in ongoing immune surveillance[22], [66]. Thus, our results raise interesting questions about how T cells suppress viral reactivation throughout the animal. The results discussed above strongly suggest that the vast majority of immune surveillance occurs in circulation or in sites exposed to the blood supply. However, there are also likely to be latently infected cells that are not exposed to the blood supply, implying that the relatively small number of inflationary cells found within the parenchyma of non-lymphoid tissues must be sufficient to control viral latency at those sites. An alternative explanation is that inflationary effector T cells are continuously migrating into non-lymphoid tissues and dying rapidly upon arrival, thereby reducing their steady-state numbers. However, in pulse/chase experiments with BrdU, we failed to find evidence that BrdU-labeled inflationary cells in the blood migrated into the parenchyma of any tissue (i.e. became preferentially protected from i.v. antibody over time) or were lost more rapidly within tissue parenchyma compared to the blood (not shown). Further experiments will be needed to address the virus-T cell dynamics at sites that are not accessible to the blood supply.

In humans, CMV infection and immunity parallel what is seen in MCMV infected mice in many ways. HCMV-specific CD8s accumulate in the blood but not the lymph nodes and turnover with similar kinetics as the inflationary populations in mice [53], [80], suggesting that a similar mechanism may sustain memory inflation in both hosts. Moreover, similar cell types and organs are infected by both viruses and it is apparent that both myeloid and non-hematopoietic cells can harbor latent HCMV and MCMV DNA[1]–[17]. There is also a host of data suggesting that endothelial cells are at least one major non-hematopoietic site of viral latency in both humans and mice. Indeed, MCMV DNA has been detected in the endothelial cells of multiple organs[1] and HCMV DNA has been found in the vessel walls of major arteries (reviewed in [3]). This, combined with our work presented here, would suggest a major role for antigen-presentation by endothelial cells in both humans and mice.

Collectively, our work suggests a new model of CMV-driven memory inflation in which immune surveillance mostly occurs in circulation, and a large proportion of newly produced effector T cells have responded to viral antigen on latently infected cells that are accessible to the blood supply. Our work may have important implications for the development of CMV-based vaccine vectors since additional measures may be needed to ensure that blood-borne T cells migrate out of the vasculature and into the desired target tissues. However, it will be exciting to learn whether endothelial cells are the primary source of viral antigen that sustains inflationary T cells and whether other infections that sustain large numbers of effector T cells (e.g. parvoviruses and adenoviruses[33]–[39]) also depend on antigen recognition within circulation.

## Methods

### Ethics statement

All animal work was performed in accordance with NIH guidelines and the Animal Welfare Act. The Thomas Jefferson University Office of Animal Resources has full accreditation from the Association for Assessment and Accreditation of Laboratory Animal Care (AAALAC). The experiments were approved by the Institutional Biosafety Committee and the Institutional Animal Care and Use Committee at Thomas Jefferson.

### Mice and Infections

Mice were purchased from Jackson Laboratory and bred in house for use in all experiments. C57BL/6 mice were used for all direct infections. For adoptive transfer experiments, CD45.1 congenic mice (B6.SJL-*Ptprc^a^ epc^b^*/BoyJ) and OT-Is on a B6 background (C57BL/6-Tg(TcraTcrb)1100Mjb/J) were used (see below). All mice were infected i.p. with 2×10^5^ plaque forming units (pfu) of virus and were considered chronically infected after 3 months. Experiments were carried out with the K181 strain of MCMV (kindly provided by Ed Mocarski) except where indicated. All viruses were grown and titered on M2-10B4 cells as described[81], except for the ΔgL virus (Figure 2) which was produced on gL-3T3 cells as described previously[50].

### Adoptive transfer experiments

For experiments shown in Figure 1F–G, mice were seeded with small numbers of congenic OT-Is and subsequently infected with MCMV-SL8-015, as previously described[56]. After more than 3 months post infection, splenocytes containing OT-Is were harvested and transferred into congenic recipients that had been infected (>3 months previously) with either wild-type MCMV (lacking the cognate antigen) or MCMV expressing the cognate SIINFEKL peptide (either MCMV-SL8-015 or K181-OVA). Mice received ∼2×10^7^ total splenocytes.

### BrdU treatment/FTY720 treatment

For the long-term BrdU pulse (Figure 1), mice were injected i.p. with 1mg of BrdU (Sigma) then subsequently provided with 0.8 mg/ml BrdU in their drinking water for 3 or 7 days. For the short-term BrdU pulse (Figure 2, 3 and 4 and Figure S2), mice were injected i.p. with 1 mg of BrdU. BrdU incorporation was assayed using the BD Biosciences Flow kit followed by FACS analysis. For short term FTY720 treatment (Figure 3), mice were injected i.p. with FTY720 (Cayman Chemical Company) at a dose of 1 mg/kg body weight on days 0, 2, 4 and 6. Cells were analyzed one day after the final FTY720 injection. For long term FTY720 treatment (Figure 4), mice were treated with FTY720 in their drinking water for 5 weeks at a concentration of 3.3 µg/ml. Water containing FTY720 was replaced every other day.

### Lymphocyte isolation

For analyses of T cells in the blood of living mice, peripheral blood was harvested from the retro-orbital sinus. Alternatively, blood was harvested from the chest cavity at sacrifice after cutting the pulmonary vein. For isolation of lymphocytes from organs, mice were sacrificed, the pulmonary vein was cut, blood was harvested and then mice were immediately perfused with approximately 20 ml PBS containing 1 U/ml heparin. Perfusion invariably resulted in visible evidence that blood was removed from all tested organs. Lymphocytes in the spleen and lymph nodes were isolated by passing through a 70 µm cells strainer to achieve a single cell suspension. Protocols to isolate non-lymphoid organ localized lymphocytes were adapted from Zhang et al [82] and Mega et al [83]. In brief, the liver, lungs, salivary gland, mammary gland, kidney and female reproductive tract were either minced with scissors or dissociated with the gentle MACS dissociator (Miltenyi Biotec). Livers were incubated at 37°C for 1 hour in digestion media containing 0.5 mg/ml collagenase type IV (Sigma), 5 mM CaCl_2_, 50 µg/ml DNase I (Roche), and 10% FBS in RPMI 1640 with L-glutamine (Cellgro). Lungs, salivary glands, mammary glands, kidneys and female reproductive tracts were incubated at 37°C for 1-1.5 hours in digestion media containing 1 mg/ml collagenase type IV, 5 mM CaCl_2_, 50 µg/ml DNase, and 10% FBS in RPMI. To isolate lymphocytes, liver, kidney and female reproductive tract homogenates were suspended in 40% Percoll (Sigma) and overlayed on top of a 70% Percoll layer, (each prepared in RPMI without serum). Salivary glands were suspended in 40% Percoll and overlayed on top of a 75% Percoll layer. Suspensions were centrifuged at 600×g for 25 minutes. Lung and mammary gland homogenates were suspended in 40% Percoll and centrifuged directly at 600×g for 25 minutes. Lymphocytes were isolated from the 70/40 interface, the 75/40 interface or the pellet respectively.

### Antibodies, tetramer staining and FACS analysis

MHC-tetramers loaded with peptides derived from M38, IE3, M57 and M45 were produced at the NIH tetramer core facility (http://tetramer.yerkes.emory.edu/) and used to identify Ag-specific T cells as described previously[43]. Phenotypic analysis was performed with the following antibodies: CD8α (clone 53–6.7), CD44 (clone IM7), CD62L (clone MEL-14), CD127 (clone A7R34), KLRG1 (clone 2F1), Ki67 (clone B56), and BrdU (clone 3D4). For identifying Ki67-positive and BrdU-labeled cells, lymphocytes were fixed and permeabilized using the BrdU Flow Kit from BD Biosciences using the recommended protocol. For adoptive transfers, OT-Is were distinguished from host cells by staining for congenic markers CD45.1 (clone A20) and CD45.2 (clone 104) and the TCR Vα2 chain (clone B20.1). All antibodies were purchased from Biolegend or BD Biosciences. Cells were analyzed on an LSR II flow cytometer (BD Biosciences) and using FlowJo software (TreeStar, Ashland, OR, USA).

### Intravenous antibody injection

Intravenous antibody injection was used to distinguish between vasculature-localized and parenchyma-localized CD8 T cells as described previously[62]–[64]. Briefly, mice were injected i.v. with 3 µg Brilliant Violet 421-labeled anti-CD8α antibody (clone 53–6.7) and sacrificed 3 minutes later. After perfusion, harvested organs were digested with collagenase as described above in the presence of 60 µg/ml unlabeled CD8α antibody. Isolated cells were stained with labeled anti-CD8β antibody (clone 53–5.8) and other phenotypic markers.

### Immunofluorescent microscopy

To confirm the sites of i.v. staining, mice were injected with APC labeled CD8α as described above. Isolated spleen, liver, lung and mediastinal lymph nodes were frozen in OCT and sectioned using a cryostat. Sections were fixed with cold acetone for 10 minutes and then stained with antibodies specific for B220 (clone RA3-6B2), F4/80 (clone BM8), CD31 (clone 390), CD45.2 (clone 104) and CD8β (clone YTS156.7.7) and co-stained with DAPI (Prolong Gold antifade – Life Technologies). All antibodies were purchased from Biolegend. Images were generated with the LSM 510 Meta confocal laser scanning microscope (Carl Zeiss) and the LSM image browser software (Carl Zeiss).

## Supporting Information

Figure S1 **Gating strategy for FACS analysis of MCMV-specific CD8 T cells.** (A) In all experiments, total cells (top left), singlets (top right) and CD8+ (bottom left) populations were gated before selection of antigen specific populations. Antigen specific CD8+ T cells were identified by staining with peptide loaded MHC-tetramers labeled with APC or PE (bottom right). (B) Gates distinguishing expression of KLRG1 and CD127 were set on all CD8s (left), and then applied to antigen-specific populations (middle and right). (C) Gates for BrdU-positive cells were set using a sample stained with an isotype control antibody (left), which was then applied to antigen-specific populations (middle and right). (D) Ki67-expressing CD8 T cells were gated within total CD8s (left) and gates were applied to antigen-specific populations (middle and right). (E) For some experiments, mice were injected with 1 mg of BrdU 16 hours before sacrifice. Cells co-staining for BrdU and Ki67 were gated among all CD8s (right) and antigen-specific populations (middle and right) as shown.
(TIFF)

Figure S2 **Effector-phenotype T cells turnover continuously during MCMV-induced memory inflation.** IE3-specific T cells accumulate and express an effector phenotype outside of lymph nodes. Shown is the frequency (A) and phenotype (B) of IE3-specific CD8s in the indicated organs in the mice described for Figures 1B and C (n = 12, CLN =  cervical lymph nodes, MLN = mediastinal lymph nodes). (C) Stable maintenance of inflationary populations over time. Shown is the total frequency of M38- and IE3-specific CD8s in the blood over time for the experiment described in Figures 1D and 1E. (D) Cohorts of mice that had been infected with K181 MCMV more than 3 months previously, were injected i.p. with 1 mg of BrdU and sacrificed 16 hours, 1 week, 3 weeks or 6 weeks later. To account for mouse-to-mouse variation in the BrdU incorporation, the frequency of BrdU-labeled M38-specific T cells in each organ at each time point, was normalized to the frequency measured in the blood within the same mouse 16 hours post BrdU injection. Shown is the normalized proportion of M38-specific T cells that were BrdU-labeled in the blood, liver, lungs and spleen over time. Data is pooled from two independent experiments (n = 4–7 for each time point) (E) Phenotype of the total inflationary population is stable over time. (E) Shown is the phenotype of all M38-specific T cells (F, left) BrdU-positive IE3-specific T cells or (F, right) total IE3-specific T cells at the indicated time points after BrdU pulse for the experiment shown in Figure 1E. As in Figure 1E, any data points with fewer than 25 labeled IE3-specific T cells were excluded from the analysis at that time point. Statistical significance was determined by comparing the proportion of BrdU-labeled cells expressing an effector phenotype relative to the week 0 time point. Statistical significance was measured by paired student' t-tests (*p<.05, **p<.01, ***p<.001). Error bars represent the standard error of the mean. (G) MCMV transcripts are undetectable during chronic infection. Shown are the results of the nested RT-PCR for IE1 (top) and β-actin (bottom) transcripts performed on cDNA extracted from mediastinal lymph nodes. Each lane represents an individual mouse that had been infected for 3 days (Lane 1) or more than 3 months (Chronic 1-3). cDNA from the lymph nodes of a naïve mouse and H_2_O serve as negative controls.
(TIFF)

Figure S3 **Systemic division of inflationary CD8s.** Ki67 expression alone reveals an elevated frequency of division in draining lymph nodes. Shown is the average frequency of Ki67 expression in M38-specific (left), IE3-specific (middle) and M45-specific (right) T cells in the indicated organs more than 3 months after (A) K181 MCMV infection (n = 12) or (B) ΔgL-MCMV infection as in Figure 2D (n = 8). (C) Shown is the phenotype of Ki67 expressing T cells from the populations shown in “B”. Error bars represent the standard error of the mean. Statistical significance was measured by a paired student's t-test (*p<.05, **p<.01).
(TIFF)

Figure S4 **FTY720 treatment does not alter the maintenance of memory inflation.** (A) Inflationary populations express low levels of CD62L. Representative FACS plots show expression of CD44 and CD62L on M38-specific (left) and IE3-specific (right) cells in the blood more than 3 months post infection, but prior to any FTY720 treatment (as in Figure 3). (B–D) The IE3-specific T cell population is stable in the blood of mice during prolonged FTY720 treatment. Shown are the IE3-specific populations from the experiment illustrated in Figure 4 indicating (B) the number of IE3-specific CD8s per milliliter of blood, (C) the percentage of blood-localized IE3-specific CD8s expressing the effector phenotype (KLRG1^pos^, CD127^low^), and (D) the frequency of Ki67-positive IE3-specific CD8s in the blood before and during the time course in treated mice (top) and controls (bottom). Error bars represent the standard error of the mean (n = 5 per group). Statistical significance was measured by paired (B–D) student's t-tests (*p<.05, **p<.01, ***p<.001).
(TIFF)

Figure S5 **Intravenous staining reveals cells that are exposed to the blood supply.** Mice infected with K181 MCMV for more than 3 months were injected with APC-labeled anti-CD8α antibody to identify T cells exposed to the blood supply. The localization of staining was confirmed with immunofluorescent staining of tissue sections. (A) In the spleen, CD8s labeled with injected antibody (white) were localized to the red pulp (identified by F4/80 expressing macrophages-shown in red) and were not found in the white pulp (delineated by B220 shown in green). The enlarged inset (1) shows overlaid lines between the red pulp and the white pulp and the arrow indicates one of several i.v. labeled CD8s in the red pulp. (B) Staining of lung sections revealed that all CD8s labeled by injected antibody (shown in white – marked by arrows in enlarged images of insets 2 and 3) co-localized with vasculature (identified by CD31 expression - red), while aggregates of lymphocytes (identified by CD45.2 expression – green) and cells in the airways that were outside the vasculature remained unlabeled. Note that many of the cells in the CD45+ aggregate (inset 3) were identified as T cells in a serial section (not shown). (C) Within mediastinal lymph nodes, i.v. stained CD8s (shown in white – marked by arrows in the enlarged images of inset 4) were only found in association with the lymph node vasculature (CD31+, shown in red) while CD8s outside of the vasculature (identified by CD8β expression – green) were unlabeled by the i.v. antibody. (D) In the liver, T cells that were labeled with i.v. injected anti-CD8α (shown in white – marked by arrows in inset 5 and the enlarged images) were found throughout the liver in structures consistent with the liver sinusoids. Staining of these structures of anti-CD31 (red) was variable and generally faint. In all cases, DAPI staining in the nucleus is shown in blue.
(TIFF)

Figure S6 **Inflationary cells shielded from the blood do not have the typical inflationary phenotype or immunodominance.** Mice infected with K181 MCMV for more than 3 months were injected with fluorochrome labeled anti-CD8α antibody to identify T cells exposed to the blood supply. (A) Shown is the mean frequency of MCMV-specific cells labeled by i.v. staining and co-stained with the indicated tetramer in the indicated organs. (B) As in Figure 6B and C, shown is the mean frequency of IE3-specific cells within the labeled or unlabeled fraction of the indicated organs that express (B) an effector phenotype (KLRG1^pos^, CD127^low^) or (C) a memory phenotype (KLRG-1^neg^, CD127^pos^). Data is pooled from three independent experiments (n = 12). (D) Absolute numbers of IE3-specific and M57-specific in the labeled fraction (left panel) and unlabeled fraction (right panel) in the indicated organs (n = 15). These numbers were used to calculate the ratios shown in Figure 7B. Error bars represent the standard error of the mean. Statistical significance was measured by paired student's t-test (*p<.05, **p<.01, ***p<.001, **** p<.0001).
(TIFF)

Figure S7 **Effector phenotype inflationary CD8s overwhelmingly localize to the blood exposed compartment.** Mice infected with K181 MCMV for more than 3 months were injected with fluorochrome labeled anti-CD8α antibody to identify T cells exposed to the blood supply. The distribution of effector phenotype or memory phenotype M38-specific cells was determined by calculating the average number of these cells in labeled and unlabeled compartment of the spleen, liver, lung, cervical and mediastinal lymph nodes, kidney, female reproductive tract, salivary gland, and mammary gland (n = 7−15). Shown is the distribution of all (left) or unlabeled (right) effector phenotype M38-specific T cells (A) or memory phenotype M38-specific T cells (B). The blood-localized fractions for each organ are displayed in red and the unlabeled fractions are displayed in blue. The numbers represent the percentage of cells at each site out of the total tetramer specific population.
(TIFF)
