# Supplementary figures and images for: Systemic Hematogenous Maintenance of Memory Inflation by MCMV Infection
Source: PLoS Pathog. 2014 Jul 3;10(7):e1004233. doi: 10.1371/journal.ppat.1004233 (PMC4081724; doi:10.1371/journal.ppat.1004233)

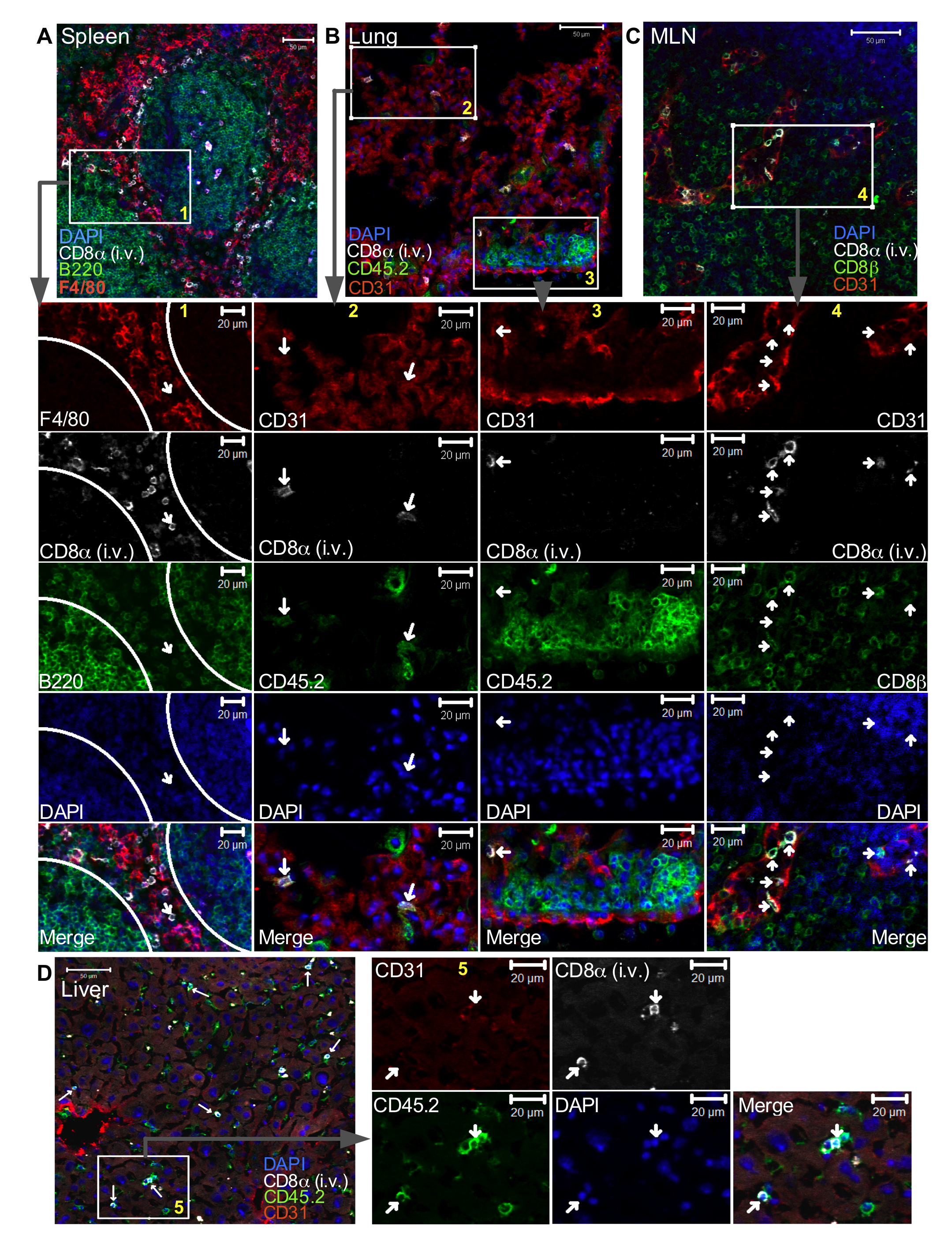

Supplement: Figure S5 — Intravenous staining reveals cells that are exposed to the blood supply. Mice infected with K181 MCMV for more than 3 months were injected with APC-labeled anti-CD8α antibody to identify T cells exposed to the blood supply. The localization of staining was confirmed with immunofluorescent staining of tissue sections. (A) In the spleen, CD8s labeled with injected antibody (white) were localized to the red pulp (identified by F4/80 expressing macrophages-shown in red) and were not found in the white pulp (delineated by B220 shown in green). The enlarged inset (1) shows overlaid lines between the red pulp and the white pulp and the arrow indicates one of several i.v. labeled CD8s in the red pulp. (B) Staining of lung sections revealed that all CD8s labeled by injected antibody (shown in white – marked by arrows in enlarged images of insets 2 and 3) co-localized with vasculature (identified by CD31 expression - red), while aggregates of lymphocytes (identified by CD45.2 expression – green) and cells in the airways that were outside the vasculature remained unlabeled. Note that many of the cells in the CD45+ aggregate (inset 3) were identified as T cells in a serial section (not shown). (C) Within mediastinal lymph nodes, i.v. stained CD8s (shown in white – marked by arrows in the enlarged images of inset 4) were only found in association with the lymph node vasculature (CD31+, shown in red) while CD8s outside of the vasculature (identified by CD8β expression – green) were unlabeled by the i.v. antibody. (D) In the liver, T cells that were labeled with i.v. injected anti-CD8α (shown in white – marked by arrows in inset 5 and the enlarged images) were found throughout the liver in structures consistent with the liver sinusoids. Staining of these structures of anti-CD31 (red) was variable and generally faint. In all cases, DAPI staining in the nucleus is shown in blue. (TIFF) [file ppat.1004233.s005.tiff]

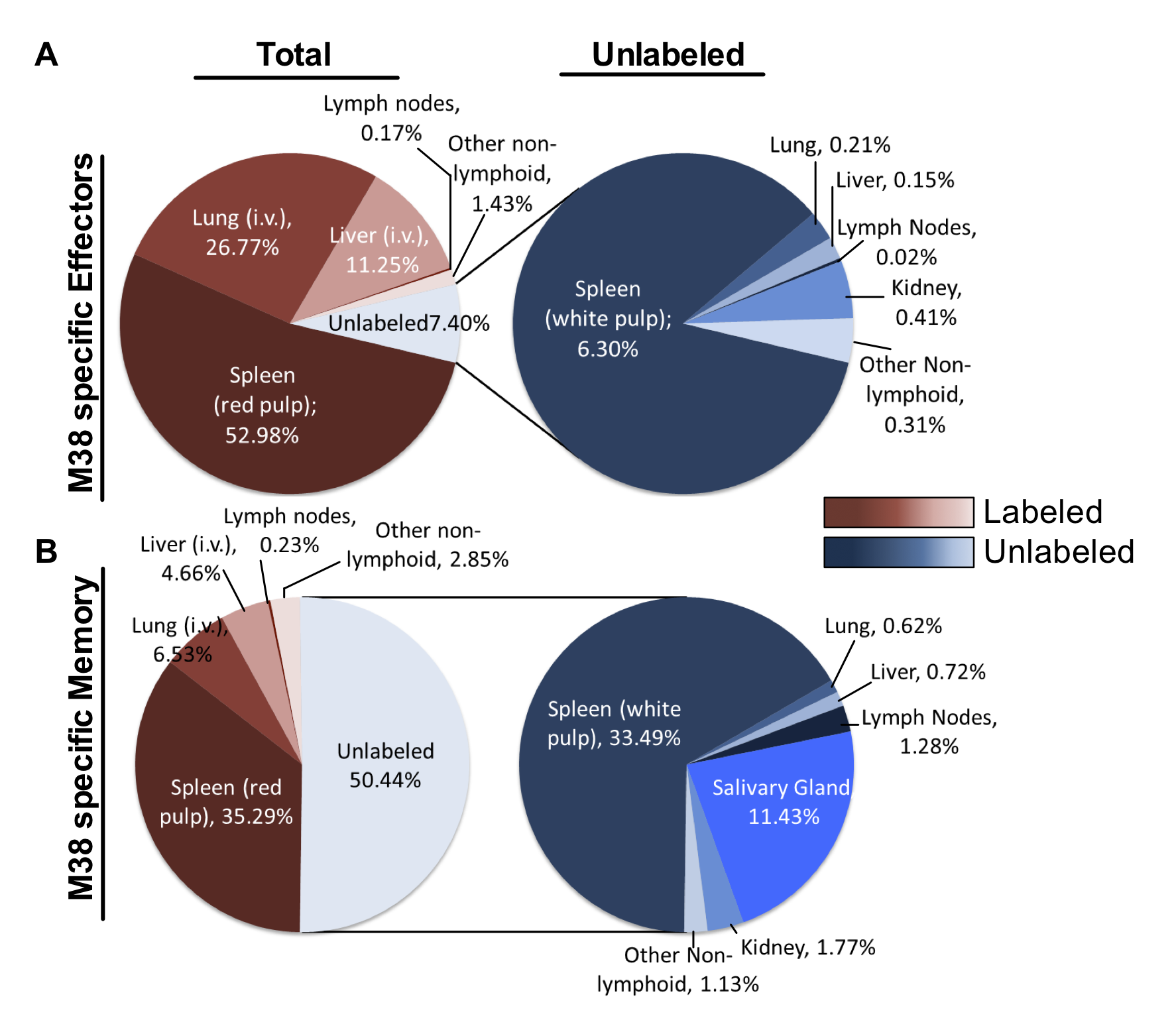

Supplement: Figure S7 — Effector phenotype inflationary CD8s overwhelmingly localize to the blood exposed compartment. Mice infected with K181 MCMV for more than 3 months were injected with fluorochrome labeled anti-CD8α antibody to identify T cells exposed to the blood supply. The distribution of effector phenotype or memory phenotype M38-specific cells was determined by calculating the average number of these cells in labeled and unlabeled compartment of the spleen, liver, lung, cervical and mediastinal lymph nodes, kidney, female reproductive tract, salivary gland, and mammary gland (n = 7−15). Shown is the distribution of all (left) or unlabeled (right) effector phenotype M38-specific T cells (A) or memory phenotype M38-specific T cells (B). The blood-localized fractions for each organ are displayed in red and the unlabeled fractions are displayed in blue. The numbers represent the percentage of cells at each site out of the total tetramer specific population. (TIFF) [file ppat.1004233.s007.tiff]
